# Resonant Rectifier ICs for Piezoelectric Energy Harvesting Using Low-Voltage Drop Diode Equivalents

**DOI:** 10.3390/s17040901

**Published:** 2017-04-19

**Authors:** Amad Ud Din, Seneke Chamith Chandrathna, Jong-Wook Lee

**Affiliations:** Department of Electronics Engineering, Information and Communication System-on-Chip (SoC) Research Center, Kyung Hee University, Yongin 17104, Korea; ammad@khu.ac.kr (A.U.D.); b13chamith@gmail.com (S.C.C.)

**Keywords:** AC-DC converters, energy harvesting, piezoelectric, rectifier

## Abstract

Herein, we present the design technique of a resonant rectifier for piezoelectric (PE) energy harvesting. We propose two diode equivalents to reduce the voltage drop in the rectifier operation, a minuscule-drop-diode equivalent (MDDE) and a low-drop-diode equivalent (LDDE). The diode equivalents are embedded in resonant rectifier integrated circuits (ICs), which use symmetric bias-flip to reduce the power used for charging and discharging the internal capacitance of a PE transducer. The self-startup function is supported by synchronously generating control pulses for the bias-flip from the PE transducer. Two resonant rectifier ICs, using both MDDE and LDDE, are fabricated in a 0.18 μm CMOS process and their performances are characterized under external and self-power conditions. Under the external-power condition, the rectifier using LDDE delivers an output power *P*_OUT_ of 564 μW and a rectifier output voltage *V_RECT_* of 3.36 V with a power transfer efficiency of 68.1%. Under self-power conditions, the rectifier using MDDE delivers a *P*_OUT_ of 288 μW and a *V_RECT_* of 2.4 V with a corresponding efficiency of 78.4%. Using the proposed bias-flip technique, the power extraction capability of the proposed rectifier is 5.9 and 3.0 times higher than that of a conventional full-bridge rectifier.

## 1. Introduction

There is increasing demand for autonomous sensing devices, deployed in various applications, such as medical, healthcare, and environmental monitoring [1]. To enable uninterrupted data gathering from a large population of sensing devices, e.g., the Internet of Things (IoT), a long lifetime is critical. Although there are continued innovations in battery capacity, battery lifetime is still finite. To extend the lifetime of sensing devices, energy can be acquired from ambient sources. There exist various sources from which energy can be extracted, including thermal, solar, vibrations, and wind. Among those energy sources, vibrations can provide a relatively large amount of energy through highly-efficient piezoelectric (PE) transducers.

The equivalent electrical model of a PE transducer is represented as a sinusoidal current source *I_P_(t)* = *I_P_*sin(*ω_p_t*) in parallel with a capacitor *C_p_* and a resistor *R_p_*, where *ω_p_* = 2*πf_p_* is the angular frequency. In general, *R_p_* is very large during low-frequency transducer operation, and the open-circuit voltage can be expressed as *V_p_* = *I_P_/ω_p_C_p_*. The output of a PE transducer is alternating current (AC) and, thus, needs conversion to direct current (DC). The commonly used AC-DC converters are full-bridge rectifiers (FBRs) and voltage doubler rectifiers (VDRs). Both FBR and VDR deliver a similar maximum output power when ideal diodes are used [2]. The operation of an FBR is well understood, which provides current for every half-cycle only after charging *C_p_* to ±(*V_RECT_* + 2*V_D_*). Here, *V_RECT_* is the rectified output voltage and *V_D_* is the diode voltage drop. The VDR provides current to the output only during the positive half-cycle. In the negative half-cycle, a diode in parallel with the PE transducer provides a path to discharge *C_p_* to the ground. During the positive half-cycle, *I_P_* only needs to charge *C_p_* from −*V_D_* to (*V_RECT_* + *V_D_*) before current can flow into the output.

In the rectifier, a commonly used figure-of-merit is the power transfer efficiency, which is defined as the ratio of the output power *P*_OUT_ to the input power *P*_IN_, which can be delivered by the PE transducer. Rectifying a low voltage from a PE transducer may induce significant power losses due to the diode voltage drop. An effective way to increase the efficiency of the rectifier is (1) reducing the diode’s voltage drop; and (2) increasing the input voltage by implementing the bias-flip strategy.

**Figure 1:**
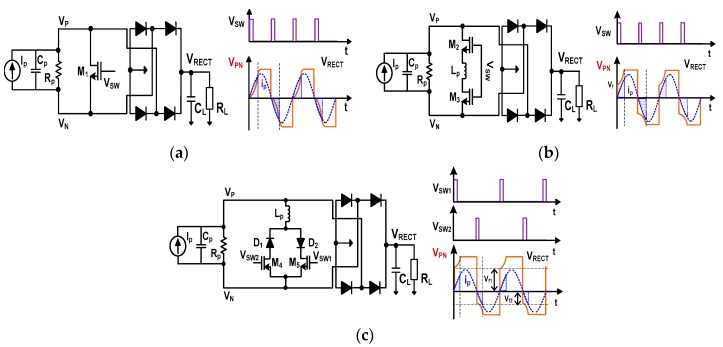
Schematics of the rectifiers for piezoelectric energy harvesting utilizing (**a**) switch-only; (**b**) bias-flip using an inductor; and (**c**) bias-flip using two switches and diodes with an inductor. The output load includes *C_L_* and *R_L_*.

Figure 1 shows several reported techniques for improving the efficiency [2,3,4,5,6]. The switch-only rectifier is introduced to reduce the power to charge *C_p_* (not delivered to output) during the negative cycle of the VDR. In this approach, a switch M_1_ is shunted across the PE transducer, as shown in Figure 1a. The purpose of this switch is to discharge *C_p_* instantaneously when *I_P_* crosses zero. Since the switch is on at the zero crossing, the initial voltage to charge *C_p_* starts from 0 rather than −*V_D_*. This modification reduces the charge which is not delivered to the output and increases the extracted power.

During the period when *C_p_* is charged, however, there is still a large portion of *I_P_* that is not delivered to the output. The highlighted portion of *I_P_*, shown in Figure 1a, indicates the portion of *I_P_*, which is used to charge *C_p_* from 0 to ±(*V_RECT_* + 2*V_D_*). To reduce the charge, the work in [2] introduces a bias-flip technique, shown in Figure 1b. An inductor *L_P_* is shunted across the PE transducer through the switches realized with M_2_ and M_3_. When *I_P_* crosses zero, the pulse signal *V_SW_* briefly turns M_2_ and M_3_ on. At this time, the resonant loop formed by *C_p_* and *L*_P_ flips the voltage, *V_PN_* = *V_P_* − *V_N_* across the transducer. Then, the charging starts from the flip-voltage *V_f_* rather than from 0 V. Since flipping reduces the amount needed to charge *C_p_* from 0 to *V_f_*, this technique increases the extracted power. However, we note that there exists power loss from the voltage drop of the two switches. In addition, the body diode of switches may be conducting for a large *V_PN_* (This observation also applies to M_1_ in Figure 1a. To reduce the voltage drop, current paths for positive and negative cycles are split [4,5,6]. Sharing a single inductor *L_P_* as shown in Figure 1c, this approach provides two branches for the bias-flip using two diodes and two shunt switches. The transducer voltage is flipped alternatively through two paths. Then, the voltage drop by the switch is reduced from two to one, but with an additional diode voltage drop. The drawback of this approach is that the two bias-flip voltages, *V_f_*_1_ and *V_f_*_2_ for positive and negative cycles, respectively, are different, i.e., *V_f_*_1_ ≠ *V_f_*_2_. This is because the impedance at *V_P_* and *V_N_* seen from the flipping path is different, and we observe asymmetric flipping in the waveform. The asymmetry results in fluctuation of the extracted power and increased output ripple.

The diode voltage drop *V_D_* existing in the rectifier loop is the third reason for the low efficiency. The *V_D_* drop can be reduced by adding a bias voltage between the gate and drain terminal of a transistor [7]. To generate the bias voltages for a multi-stage rectifier, an extra bias distributor is required, which increases circuit complexity and the losses associated with it. In [3], an active diode, based on an op-amp with a pre-set DC offset, is used to reduce *V_D_* and the leakage current. Another method to reduce *V_D_* is by using a comparator-controlled switch [8]. This approach requires approximately one threshold voltage *V_TH_* plus two overdrive voltages to power up, limiting the input voltage for start-up to 1.2 V using a 0.35 µm CMOS process. Usually, the comparator is powered up from the output storage capacitor. If there is not enough voltage to power up, the comparator will not be readily activated.

Herein, we propose two resonant rectifiers using low-voltage drop diode equivalents to overcome the limitations that exist in previous studies. We propose two diode equivalents, a minuscule-drop-diode equivalent (MDDE) and a low-drop-diode equivalent (LDDE), which effectively reduces the *V_D_* of the rectifying stage. The diode equivalents are efficiently combined with a symmetric and low-loss resonant loop to realize the bias-flip technique. Harnessing MDDE and LDDE, two resonant rectifier integrated circuits (ICs) having self-startup capabilities are designed. To improve the efficiency under the self-power condition, the rectifier using MDDE includes synchronous bootstrap pulse generators (SBPGs). The SBPG provides boosted bias-flipping pulses that are synchronized with the frequency of the PE transducer. Two resonant rectifier ICs using both MDDE and LDDE, are fabricated in a 0.18 μm CMOS process. The rectifier using LDDE shows measured *P*_OUT_ of 564 μW under an external-powered condition with a corresponding efficiency of 68.1%. The rectifier using MDDE shows enhanced efficiency under the self-powered condition. It delivers a *P*_OUT_ of 288 μW with a corresponding efficiency of 78.4% and this result compares favorably with results from previous works.

## 2. Design

For an energy harvester, the capability for self-startup is one of the critical functions and several techniques have been reported [9,10,11,12]. In [9], the authors introduce a cold startup technique using a transformer. For high voltage boosting, this approach needs a transformer with a large turn ratio, which can increase the overall size of the harvester. In [10], the authors propose a mechanical switch that provides an instant power jerk to kick start the harvester. In [11], the authors present a low-voltage startup technique using a *V_TH_*-tuned oscillator and a capacitor pass-on technique. Although a low startup voltage of 95 mV is achieved, the drawback is that this approach requires external programming of a body voltage after fabrication. In [12], a charge pump with a switched body-biasing technique is presented. Since the body terminal of a transistor is connected to a high voltage when it is turned off, the reverse leakage is effectively suppressed. In these previous works, except [11], the self-startup function, which is vital to autonomous operation, is not fully supported. In this work, we embed a simple, yet efficient, approach for self-startup into the two resonant rectifiers.

### 2.1. Resonant Rectifier Using LDDE

**Figure 2:**
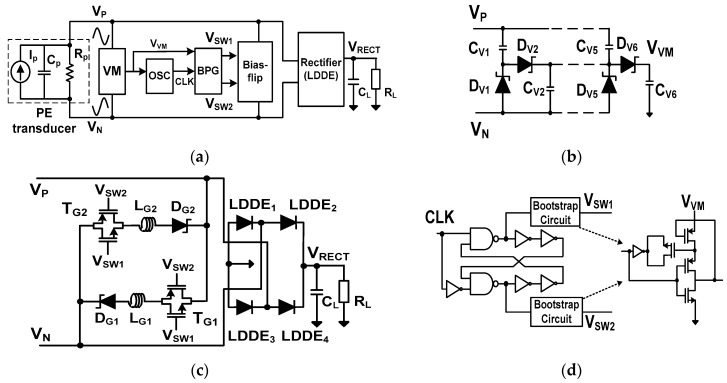
(**a**) Block diagram of the proposed resonant rectifier IC using LDDE; (**b**) voltage multiplier; (**c**) bootstrap pulse generator; and (**d**) the symmetric bias-flip circuit and rectifying stage using LDDEs.

Figure 2a shows the block diagram of the resonant rectifier IC using LDDE. The rectifier consists of a three-stage voltage multiplier (VM), an oscillator (OSC), a bootstrap pulse generator (BPG), a symmetric bias-flip circuit, and a full-bridge rectifying stage. The BPG provides the bootstrapped pulse signals *V*_SW1_ and *V*_SW2_ for the bias-flip circuit. The clock (CLK) signal for the BPG is generated by the OSC with a frequency that can be tuned using a ring oscillator. The supply voltage of the OSC is driven by the output *V*_VM_ of the voltage multiplier shown in Figure 2b. For efficient operation, the VM uses Schottky diodes (D_V1_–D_V6_) realized in a standard CMOS process, which shows a low *V_D_* of 160 mV at 1 µA [13]. Since the VM and OSC are powered from the PE transducer, the rectifier provides the self-startup function.

Figure 2c shows the schematic of the BPG which generates *V_SW_*_1_ and *V_SW_*_2_. The amplitudes of these signals are increased to almost 2*V_VM_*, which provide the high overdrive voltage needed to fully turn on the switches in the bias-flip circuit, shown in Figure 2d. The bias-flip circuit consists of Schottky diodes (D_G1_, D_G2_), inductors (L_G1_, L_G2_), and transmission (T)-gate transistors (T_G1_, T_G2_). The T-gate is used to reduce the on-resistance of the switch [14]. The size of NMOS and PMOS transistors in the T-gate are W/L = 1000 µm/0.2 µm and 2000 µm/0.18 µm, respectively, with values optimized by using a circuit simulator. When *I_P_* crosses zero, the bias-flip circuit changes the polarity of *V_PN_* using two separate paths. The positive cycle uses the path formed by T_G1_, L_G1_, and D_G1_, while the path formed by T_G2_, L_G2_, and D_G2_ is used in the negative cycle. Inductors are bulky and expensive; the bias-flip circuit shown in Figure 2d is improved to use only one inductor (see Section 2.2).

**Figure 3:**
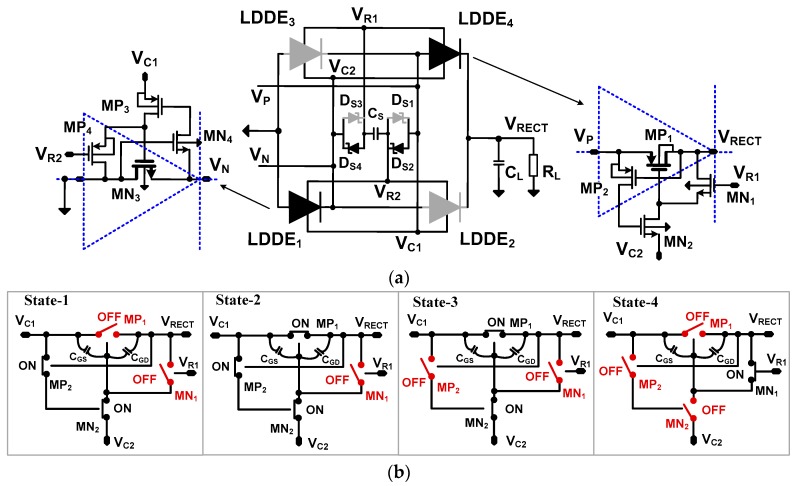
(**a**) Schematic of the rectifier loop using LDDEs; and (**b**) four operation states described using LDDE_4_.

Figure 3 shows a detailed schematic of the rectifying stage using four LDDEs. LDDE_1,3_ are NMOS-based LDDEs and the LDDE_2,4_ are PMOS-based LDDEs. When *V_P_* > *V_N_*, LDDE_1_ and LDDE_4_ conduct and form a closed loop. LDDE_4_ consists of a main transmission transistor MP_1_ and control circuit (MP_2_, MN_1_, and MN_2_). The LDDE_1_ consists of the main transistor MN_3_ and control circuit (MN_4_, MP_3_, and MP_4_). In a previous work [15], the control circuit for the LDDE is implemented using discrete bipolar junction transistors (BJTs). In this work, we remove the base current of the BJT using metal-oxide field effect transistors (MOSFETs) realized in IC technology. A voltage polarity sense circuit is formed by the diodes D_S1_–D_S4_ and *C_S_*. The circuit detects the positive (*V_P_ > V_N_*) and negative (*V_P_ < V_N_*) cycles using four terminal voltages, *V_R_*_1_, *V_R_*_2_, *V_C_*_1_, and *V_C_*_2_. Using the four voltages, the sense circuit controls the conduction of the main transistor while blocking reverse leakage current. The voltage at nodes *V_C_*_1_ and *V_C_*_2_ control the conduction of LDDE_1_ and LDDE_4_ in the positive cycle (LDDE_2_ and LDDE_3_ in the negative cycle), respectively. The voltage at nodes *V_R_*_1_ and *V_R_*_2_ blocks reverse conduction of LDDE_4_ and LDDE_1_ in the negative cycle (LDDE_3_ and LDDE_2_ in the positive cycle), respectively.

The operation of the LDDE is described using four states, shown in Figure 3b. When *I_P_* crosses zero from negative to positive, D_S2_ and D_S4_ are forward-biased forming the conducting path D_S2_ – C_S_ – D_S4_. The terminal voltages detected by the sense circuit satisfy the condition (*V_C_*_1_ = *V_P_*) > (*V_R_*_2_ = *V_P_* − *V_D_*_1_) > (*V_R_*_1_ = *V_N_* + *V_D_*_1_) > (*V_C_*_2_ = *V_N_*), where *V*_D1_ is the forward voltage drop of a diode in the sense circuit. We consider four operation states for LDDE_4_ as follows.

(1) State-1: In the positive cycle when *V*_P_ > *V*_N_, we have the condition of (*V_P_* = *V_C_*_1_) > (*V_C_*_2_ = *V_N_*). Terminal *V_C_*_2_ is connected to the negative terminal *V_N_* of the PE transducer through MN_2_. Then MP_2_ turns on and subsequently turns on MN_2_, as well. The *C_SG_* of MP_1_ is charged by *V*_PN_.
(2) State-2: The source and gate terminals of MP_1_ are approximately *V_P_* and *V_N_* + *V*_*DS,MN*2_, respectively. Here, *V*_*DS,MN*2_ is the drain-source voltage of MN_2_. The voltage at node *V_P_* keeps increasing. Then, MP_1_ begins conducting when *V_SG_* > |*V_TH_*|. The voltage across C_SG_ of MP_1_ keeps increasing and MP_1_ enters the triode from the saturation mode. Using the rectifier operation, *V*_RECT_ increases. Then, the condition (*V_PN_* − *V*_*DS,MN*2_ − |*V_TH_*|) > (*V_P_* − *V_RECT_*) allows MP_1_ to enter the triode mode, which can be written as *V_RECT_* > (*V_N_* + |*V_TH_*| + *V*_*DS,MN*2_).
(3) State-3: When the *V_SD_* of MP_1_ decreases by increasing *V*_RECT_, it turns MP_2_ off. When MP_2_ is off, C*_SG_* of MP_1_ stops charging and it maintains the *V_SG_* of MP_1_. This allows MP_1_ to continue conducting in the triode mode. When MP_2_ is off, the gate of MN_2_ has no path to conduct and, therefore, MN_2_ is kept on. This state is different from the BJT version of the LDDE [15]; the base current of a BJT provides a path to discharge while the MOSFET MN_2_ is kept on.
(4) State-4: When *i_p_* changes direction (*V_P_* < *V_N_*), the current direction in the sense circuit is reversed. Then, the terminal voltages detected by the sense circuit satisfy the condition (*V_C_*_2_ = *V_N_*) > (*V_R_*_1_ = *V_N_* − *V_D_*_1_) > (*V_R_*_2_ = *V_P_* + *V_D_*_1_) > (*V_C_*_1_ = *V_P_*). This condition turns on MN_1_. By discharging the *C_GD_* of MP_1_, MN_1_ subsequently turns off MP_1_ to prevent reverse leakage. Since *V_C_*_2_ is positive and increasing, MN_2_ is turned off, which prevents the discharging of *V_RECT_* through MN_1_.

In the case of *V_P_* < *V_N_*, LDDE_2_ and LDDE_3_ conduct. During the zero-crossing of *I_P_* from positive to negative, D_S1_ and D_S3_ are forward-biased, forming the conduction path D_S3_–C_S_–D_S1_. The operation of LDDE_2_ and LDDE_3_ follow four states in a manner similar to that described above. As other sensor-free bias-flip rectifiers, the frequency control method of the rectifier using LDDE is not adaptive to the *f_p_* of PE transducer. If *f_p_* changes by aging or other reasons, the bias-flip operation may not occur at the exact zero crossing of *I_P_*. To handle this issue, a simple yet effective frequency tracking method is implemented in the rectifier using MDDE, as explained in the next section.

### 2.2. Resonant Rectifier Using MDDE

**Figure 4:**
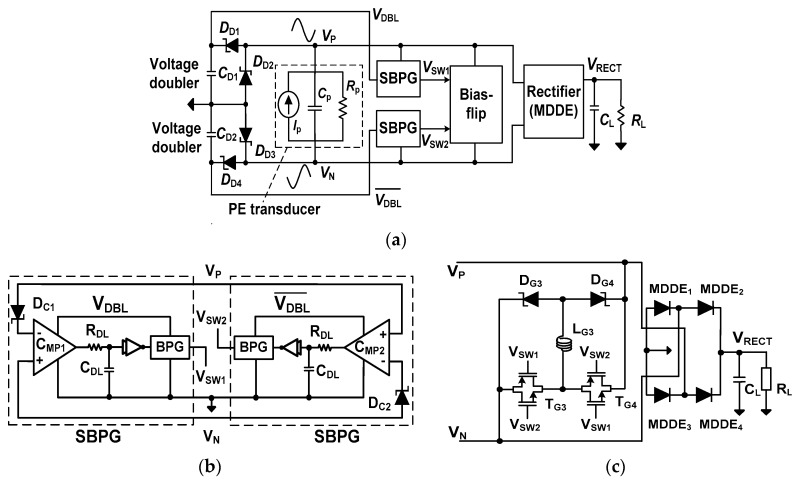
(**a**) Block diagram of the proposed resonant rectifier IC using MDDE; (**b**) synchronous bootstrap pulse generator; and (**c**) bias-flip circuit and rectifying stage using MDDE.

Figure 4a shows the block diagram of the resonant rectifier using MDDE. The rectifier IC includes two synchronous bootstrap pulse generators (SBPGs), dual voltage doublers, a symmetric bias-flip circuit, and a rectifying stage using MDDE. Instead of using VM and OSC for frequency generation, the pulse signals *V_SW_*_1_ and *V_SW_*_2_ for the bias-flip circuit are directly derived from the PE transducer. For efficient bias-flipping, the *V_SW_*_1_ and *V_SW_*_2_ are bootstrapped by the SBPG, which is driven by the output *V*_DBL_ from a voltage doubler. The dual voltage doublers are realized using the Schottky diodes D_D1_–D_D4_. Without an external power supply, the SBPG and doublers are driven by the PE transducer, realizing the self-startup function.

Figure 4b shows a schematic of the SBPG. The two SBPGs operate in a complementary manner, generating two opposite phase pulse signals, *V_SW_*_1_ and *V_SW_*_2_. In the SBPG, *V_P_* is compared with *V_N_* and the output of the comparator *C_MP_*_1,2_ drives the BPG. In this way, the voltages *V_SW_*_1_ and *V_SW_*_2_ for the bias-flip circuit are generated in sync with the *f*_p_ of the PE transducer. The comparator C_MP1,2_ is realized using a differential amplifier with a latched load for increased gain. It achieves an open-loop gain of 35 dB by consuming 770 nA. Two diodes, D_C1_ and D_C2_, are used to prevent reverse leakage current. By the BPG, the amplitudes of *V_SW_*_1_ and *V_SW_*_2_ are increased by about twice that of *V_DBL_*, which effectively flips the voltage across the nodes *V_P_* and *V_N_*.

To compensate the time delay between *V_SW_*_1,2_ and *V_PN_*, a delay line consisting of *R_DL_* and *C_DL_* is added. This allows fine tuning of the delay, which aligns the pulses *V_SW_*_1_ and *V_SW_*_2_ with the zero crossing of *I_P_*. Due to the power constraint for self-startup, the values of *R_DL_* and *C_DL_*, which depend on transducer parameters (*R_L_*, *I_P_* and *f_P_*), are externally controlled (Table 1). We perform circuit simulations and the results show that the *f_P_* from 100 to 800 Hz can be handled using this approach. The minimum delay determined by the comparator, a buffer, and BPG sets the 800 Hz as the maximum input frequency. Figure 4c shows the schematic of the bias-flip circuit and the rectifying stage using the MDDEs. To control the path for bias-flip, the Schottky diodes D_G3_ and D_G4_ are used. Using a single inductor L_G3_, two separate and symmetric paths are created for the positive (a path along T_G4_, L_G3_, and D_G3_) and negative (a path along T_G3_, L_G3_ and D_G4_) cycles. Compared to the bias-flip circuit for the rectifier using LDDE, the circuit for the MDDE is improved to use one shared inductor.

**Figure 5:**
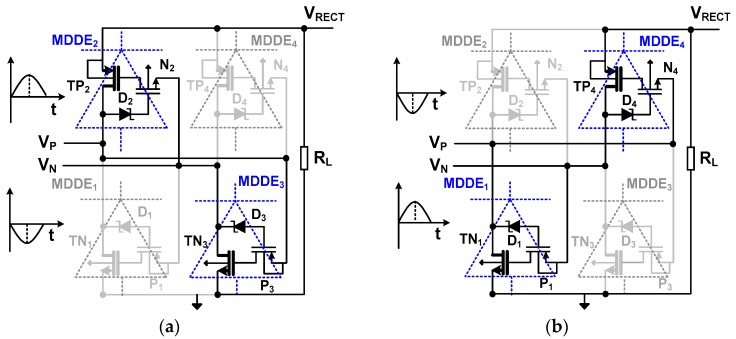
Schematic of the rectifier loop using MDDE and operation during (**a**) positive and (**b**) negative cycles.

Figure 5 shows the operation of the rectifying stage using MDDEs. The bridge-type stage consists of two PMOS and NMOS-based MDDEs. MDDE_2,4_ are PMOS-based MDDEs, where TP_2_ and TP_4_ are the main transmission transistors. MDDE_1,3_ are NMOS-based MDDEs, where TN_1_ and TN_3_ are the main transistors. The control circuit, which consists of a diode and a transistor in each MDDE, reduces the *V_D_* of the main transistor via the deep-triode mode while blocking the reverse leakage current. In the positive cycle (*V_P_* > *V_N_*), MDDE_2_ and MDDE_3_ close the loop, as shown in Figure 5a. The diode D_2_ is forward biased, which turns on N_2_. At this time, the gate of TP_2_ is connected to *V_N_* through N_2_, which turns on TP_2_. Since *V_N_* is negative, it also turns on P_3_ and D_3_. The *V*_SG_ of TP_2_ is determined by the voltage at the gate (*V_N_* + *V_DS_*_,_*_N_*_2_) and source (*V_RECT_*). With the condition (*V_RECT_* − *V_N_* − *V_DS,N_*_2_) > |*V_TH_*|, TP_2_ is turned on. The *V_GS_* of TN_3_ is determined by the voltage at the gate (*V_P_* − *V_SD,P_*_3_) and source (*V_RECT_* − *V_D,RL_*), where *V_SD,P_*_3_ and *V_D,RL_* are the source-drain voltage of P_3_ and the voltage drop across *R_L_*, respectively. With the condition (*V_P_* − *V_SD,P_*_3_ − *V_RECT_* + *V_D,RL_*) > *V_TH_*, the TN_3_ connects between *V_N_* and *V_RECT_*, closing the loop.

Next, we find the condition for TP_2_ and TN_3_ to operate in triode mode. In TP_2_, we observe that the source-drain voltage is *V_RECT_* − *V_P_* and the source-gate voltage is *V_RECT_* − (*V_N_* + *V*_*DS,N*2_). Therefore, the condition *V_PN_* >> (|*V_TH_*| + *V*_*DS,N*2_) allows deep-triode operation of TP_2_. For TN_3_, we observe that the drain-source voltage is *V_N_* − (*V_RECT_* − *V_D,RL_*) and the gate-source voltage is (*V_P_* − *V*_*SD,P*3_) − (*V_RECT_* − *V_D,RL_*). Then, the condition *V_PN_* >> (*V_TH_* + *V*_*SD,P*3_) allows deep-triode mode operation for TN_2_. In the negative cycle (*V_P_* < *V_N_*), D_2_ and D_3_ are reverse biased, and the voltage at the source terminal of N_2_ is *V_N_* > 0 (for P_3_, it is *V_P_* < 0). Therefore, both N_2_ and P_3_ are kept off and the reverse leakage through TP_2_ and TN_3_ is blocked. The operation of MDDE_1_ and MDDE_4_ in the negative cycle (*V_P_* < *V_N_*) can be similarly described, as shown in Figure 5b.

**Figure 6:**
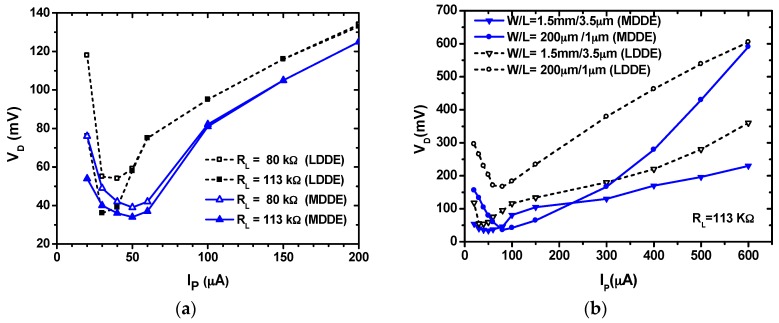
Comparison of the diode voltage drops as a function of *I_P_* for (**a**) different loads; and (**b**) periphery (W/L).

Figure 6 shows the comparison of *V_D_* of the LDDE and MDDE as a function of *I_P_*. The two *R_L_* values and the size of the main transistor are chosen to match the result in [15]. Since the control circuit does not fully turn on with small *I_P_* values, the results show that the *V_D_* of both diode equivalents increases in the small *I_P_* range from 20 to 40 µA. When *I_P_* increases above 60 µA, *V_D_* increases with *I_P_*. The LDDE has a narrow window in the *I_P_* range from 30 to 40 µA. In the case of MDDE, the *V_D_* is below 50 mV in the *I_P_* range from 30 to 60 µA. In order to investigate the performance for different PE transducer parameters, Figure 6b shows the comparison of *V_D_* of the LDDE and MDDE for the different periphery. For a PE transducer having *I_P_* = 600 µA, the *V_D_* is 230 mV and 360 mV in case of MDDE and LDDE having the same size, respectively. The results show that the MDDE shows an overall smaller *V*_D_ than that of the LDDE over a broad range of *I_P_*. The LDDE use three extra transistors to control the on-resistance of the main conducting transistor. In the case of the MDDE, the control is achieved using a transistor and a diode, which makes it simple, with a low loss.

### 2.3. Loss Calculation

**Figure 7:**
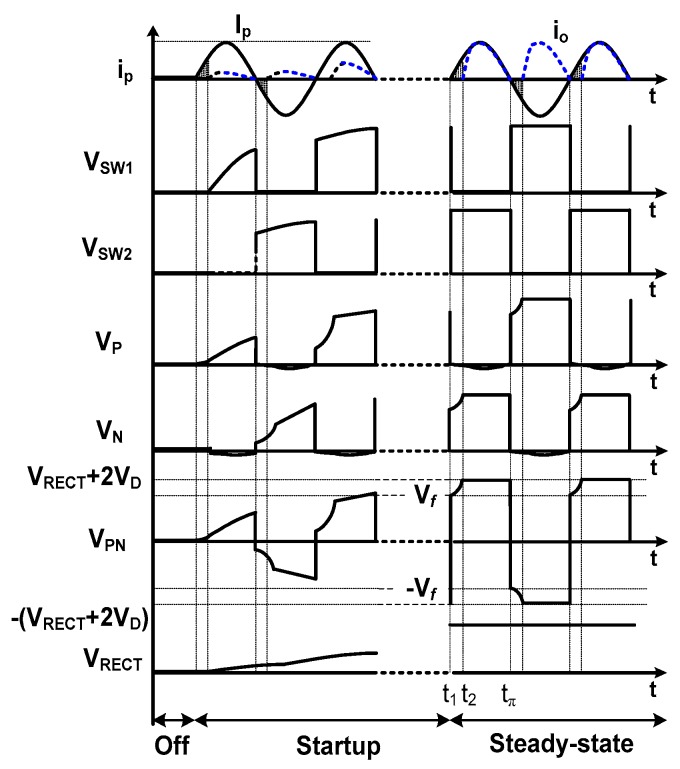
Key waveforms of the resonant rectifier.

Figure 7 shows the key waveforms of the resonant rectifier. When the PE element starts providing *I_P_*, the rectifier enters a startup state. In this state, the BPG starts generating *V_SW_*_1_ and *V_SW_*_2_, which have amplitudes that increase with *V_PN_*. A steady-state is assumed after *t*_1_. Just before time *t*_1_, *C_p_* is pre-recharged to −(*V_RECT_ +* 2*V_D_*). At time *t*_1_, *I_P_* changes direction and the bias-flip operation allows the charging of *C_p_* from *V_f_* to (*V_RECT_* + 2*V_D_*) until *t*_2_. During this period, the output current *I*_o_ starts flowing to the load. In the negative cycle, *C_p_* is discharged from −*V_f_* to −(*V_RECT_* + 2*V_D_*). The effectiveness of bias-flip is usually expressed using a flipping efficiency *η_f_*, defined as:

(1) $$\eta_{f}\;=\;\frac{V_{f}\;+\;V_{RECT}}{2V_{RECT}}$$

The amount of charge *Q_Cp_* lost due to charging *C_p_* during time interval [*t*_1_, *t*_2_] can be expressed as:

(2) $$Q_{Cp}\;=\;[V_{RECT}\;+\;2V_{D}\;-\;V_{f}]C_{p}\;=\;[V_{RECT}\;+\;2V_{D}\;-\;V_{RECT}(2\eta_{f}-1)]C_{p}$$

Next, we consider the charge lost across *R_p_* in the time interval [*t*_1_, *t*_π_]. Since *V_PN_* varies during the time interval, we consider two cases of charge losses, *Q_Rp_*_1_ during [*t*_1_, *t*_2_] and *Q_Rp_*_2_ during [*t*_2_, *t*_π_], given by:

(3) $$Q_{Rp}\;=\;Q_{Rp1}\;+\;Q_{Rp2}\;=\;\int_{t1}^{t2}\frac{V_{PN}(t)}{R_{p}}dt\;+\;\int_{t2}^{t\pi }\frac{V_{PN}(t)}{R_{p}}dt$$

During the time interval [*t*_1_, *t*_2_], *V_f_* is inverted via a bias-flip. In this period, *V_PN_*(*t*) can be obtained by integrating *I_P_*(*t*) as:

(4) $$V_{PN}(t)\;=\;\frac{1}{C_{p}}\int_{t1}^{t}I_{p}\sin\omega t\;dt\;-\;V_{f}(t_{1})\;=\;\frac{I_{p}}{\omega C_{p}}(\cos\omega t_{1}\;-\;\cos\omega t)\;-\;V_{f}(t_{1})$$

Applying the boundary conditions $V_{f}\;=\;V_{RECT}(2\eta_{f}\;-\;1)$ and *ωt*_1_ ≅ 0, Equation (4) can be expressed as:

(5) $$V_{PN}(t)\;=V_{p}(1-\cos\omega t)\;-\;V_{RECT}(2\eta_{f}\;-\;1)$$

where *V_P_* = *I_P_*/(*ω_p_C_p_*) is the open-circuit voltage. Using Equation (5), *Q_Rp_*_1_ is obtained as:

(6) $$Q_{Rp1}\;=\;\int_{t1}^{t2}\frac{V_{p}(1-\cos\omega t)\;-\;V_{RECT}(2\eta_{f}-1)}{R_{p}}dt\;≅\;\frac{V_{p}}{\omega R_{p}}(\omega t_{2}-\sin\omega t_{2})\;-\;\frac{V_{RECT}}{\omega R_{p}}(2\eta_{f}\;-\;1)\omega t_{2}$$

To find *Q_Rp_*_2_ during the time interval [*t*_2_, *t*_π_], we need the value of *ωt*_2_ and, thus, use the following relationship:

(7) $$I_{p}\sin\omega t\;=\;C_{p}\frac{dV_{p}}{dt}\;=\;\omega C_{p}\frac{dV_{p}}{d\omega t}$$

Integrating Equation (7) over the time interval [*t*_1_, *t*_2_] when bias-flipping is performed, we obtain:

(8) $$V_{p}(-\cos\omega t_{2}\;+\;\cos\omega t_{1})\;=\;V_{p}(t_{2})\;-\;V_{p}(t_{1})$$

The values of *V_p_* can be obtained from the waveform, which are $V_{p}(t_{1})\;=\;V_{f}$ and $V_{p}(t_{2})\;=\;(V_{RECT}\;+\;2V_{D})$. Then, Equation (8) can be written as:

(9) $$t_{2}\;=\;\frac{1}{\omega }\cos^{-1}(\frac{V_{p}\;-\;2V_{RECT}\;-\;2V_{D}\;+\;V_{RECT}2\eta_{f}}{V_{p}})$$

Using Equation (9), *Q_Rp_*_2_ is obtained as:

(10) $$Q_{Rp2}\;=\;\frac{\left(V_{RECT}+2V_{D}\right)}{R_{p}}(t_{\pi }\;-\;t_{2})$$

where $t_{\pi }\;=\;\pi /\omega$.

The total charge produced by the PE transducer in every cycle is given by:

(11) $$Q_{total}\;=\;2I_{p}/\omega \;=\;2C_{p}V_{p}$$

The theoretical extracted power *P*_OUT,calc_ from the PE transducer for every cycle is obtained by taking the available charge Equation (11) minus the various loss terms given by Equations (2), (6) and (10):

(12) $$\begin{array}{l} P_{\text{OUT,calc}}\;=\;2f_{p}V_{RECT}(Q_{total}\;-\;Q_{Cp}\;-\;Q_{Rp1}\;-\;Q_{Rp2})\\ =2f_{p}V_{RECT}\{[2V_{p}C_{p}\;-\;[V_{RECT}\;+\;2V_{D}\;-\;V_{RECT}(2\eta_{f}\;-\;1)]C_{p}\;-\;[\frac{V_{p}}{\omega R_{p}}(\omega t_{2}-\sin\omega t_{2})\;-\;\frac{V_{RECT}}{\omega R_{p}}\\ \left(2\eta_{f}\;-\;1)\omega t_{2}]\;-\;[\frac{V_{RECT}+2V_{D}}{R_{p}}(t_{\pi }\;-\;t_{2})]\right\} \end{array}$$

## 3. Measured Results

**Figure 8:**
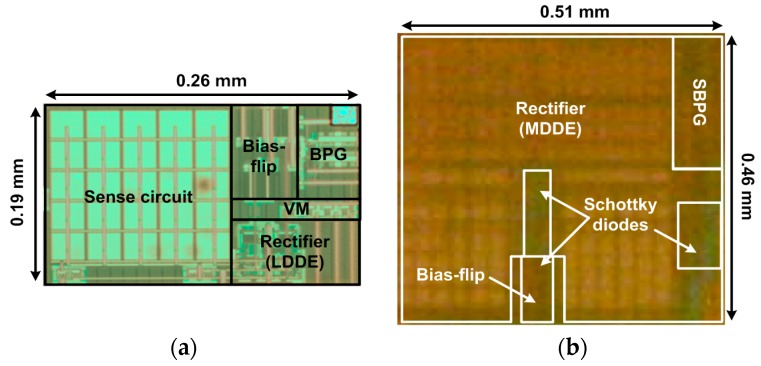
Chip micrograph of the resonant rectifier ICs using (**a**) LDDE; and (**b**) MDDE.

**Figure 9:**
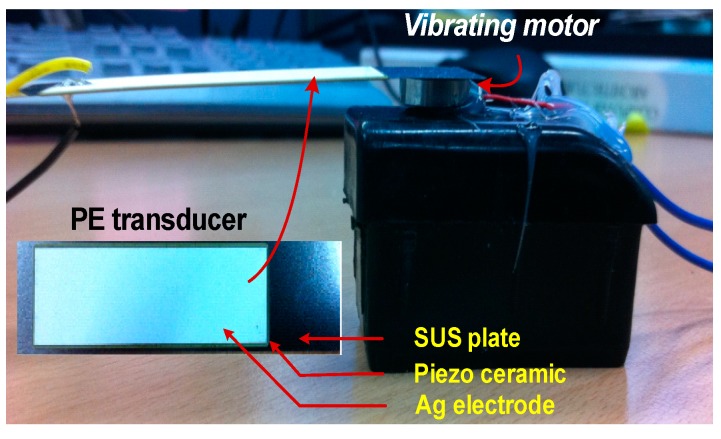
Experimental setup.

The proposed rectifiers are fabricated in a one-poly six-metal 0.18 µm CMOS process with a top 2 µm thick metal option. Figure 8 shows the chip microphotographs. The size of the rectifiers using LDDE and MDDE are 0.26 mm × 0.19 mm and 0.51 mm × 0.46 mm, respectively. Figure 9 shows the experimental setup to characterize the rectifier using the PE transducer.

The bimorph of the transducer has a thickness, a length and a width of 0.33, 75, and 20 mm, respectively [16]. Each layer consists of a stainless steel (SUS) plate, a piezo ceramic, and an Ag electrode. The PE transducer is mounted on a vibrating motor for mechanical excitement [17]. The transducer is excited using 200 Hz with an acceleration of 1.8 g. Under this condition, the theoretical optimum loading resistance of the PE transducer is 7.2 kΩ. Two wires attached to both sides of the transducer are interfaced with a test board containing the rectifiers. Table 2 shows the parameters used for the experiment. The value of the inductor for bias-flip is selected considering the trade-off between size and the Q-factor. The maximum current available from the transducer is 600 µA, which corresponds to *V_p_* = 4.34 V at 200 Hz. The equivalent circuit of a PE transducer can be represented as a mechanical spring-mass coupled to an electrical circuit. The current in the primary mechanical side corresponds to proof mass relative velocity, which is determined by such parameters as PE material, dimension, and acceleration. By the electromechanical coupling factor, which describes the effectiveness of the conversion from mechanical to electrical energies, the maximum current is determined.

**Figure 10:**
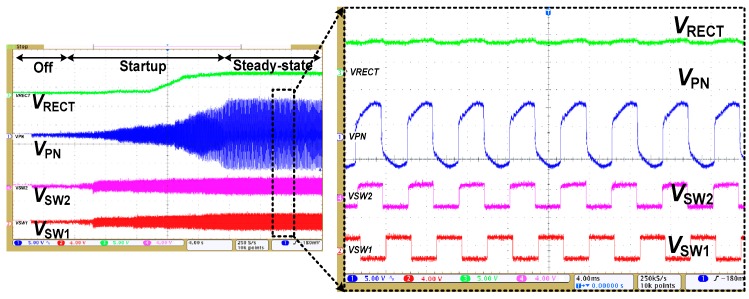
Measured waveforms of the resonant rectifier using LDDE.

Figure 10 shows the measured result of the rectifier using LDDE. It shows the input voltage *V_PN_*, bootstrap pulse signals *V_SW_*_1_ and *V_SW_*_2_, and the output *V_RECT_*. The transient during self-startup is shown in the inset. When *V_PN_* is increased, the BPG starts generating *V_SW_*_1_ and *V_SW_*_2_. Then, *V_RECT_* starts increasing and the steady-state condition is reached at about 20 s. After this time, *V_SW_*_1_ and *V_SW_*_2_ reach a value about twice that of the multiplier output and fully turn on the bias-flip circuit.

**Figure 11:**
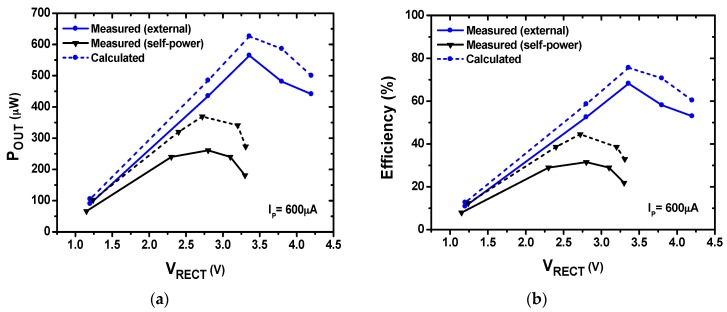
Performance of the rectifier using LDDE: (**a**) output power versus output voltage; and (**b**) power transfer efficiency versus output voltage.

Figure 11 shows the measured and calculated *P*_OUT_ and efficiency versus *V_RECT_*. We characterize the rectifier under external and self-power conditions. When it is externally powered, the VM provides power only to the BPG and the internal oscillator is turned off. The CLK frequency is supplied externally to match the *f_p_* of the PE transducer. By receiving the CLK, the BPG generates *V_SW_*_1_ and *V_SW_*_2_. The *V*_RECT_ is measured with *I_P_* = 600 µA. Since *V_RECT_* depends on *R_L_*, it is varied from 10 to 200 kΩ, searching for an optimum value. When *V_RECT_* reaches 3.36 V, the measured peak *P*_OUT_ of 564 µW is achieved with an *R_L_* of 20 kΩ. The *P*_OUT,calc_ obtained using Equation (12) is 626 µW, which indicates that the power consumed by the control circuit is 62 µW. The maximum input power which can be delivered by the PE transducer is obtained as *P_IN_* = 2 *C_p_f_p_*(*V_p_*)^2^ = 828 µW. Using the definition for power transfer efficiency = *P*_OUT_/*P_IN_*, we obtain 68.1%. Using the measured *V_f_* of 1.53 V in Equation (1), a flipping efficiency *η_f_* of 72.8% is achieved. Under the same PE input condition and with *V_D_* = 0.7 V, the maximum power that can be obtained using the FBR [2] is 190 µW. The results show that the rectifier using LDDE delivers three times higher *P*_OUT_ than that of the FBR.

Under the self-power condition, a resistive voltage divider is placed at the output of the VM. The role of the divider is controlling the supply voltage of OSC, which determines the CLK. The voltage divider is tuned so that the CLK frequency is closely matched to the *f_p_* of PE transducer. The results show a *P*_OUT_ of 261 µW with a *V_RECT_* of 2.8 V.

**Figure 12:**
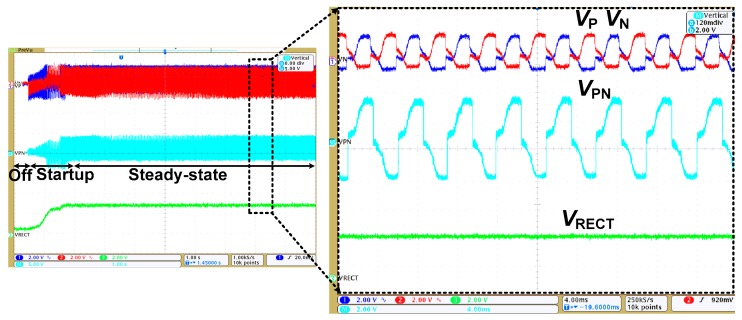
Measured waveforms of the resonant rectifier using MDDE.

**Figure 13:**
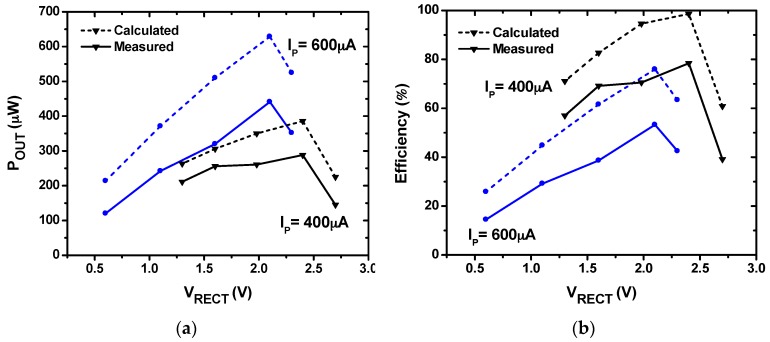
Performance of the rectifier using MDDE for two *I_P_* values: (**a**) output power versus output voltage; and (**b**) power transfer efficiency versus output voltage.

Figure 12 shows the measured waveform of the rectifier using MDDE. The result shows the initial transient waveform during self-startup. When *V_PN_* starts increasing, the SBPG generates pulses for the bias-flip. After the steady-state condition is reached at about 2 s, *V*_PN_ flips abruptly. Figure 13 shows the measured and calculated *P*_OUT_ and efficiency versus *V_RECT_* for two cases: *I_P_* = 600 µA and 400 µA. The rectifier is characterized by varying *R_L_* from 10 to 200 kΩ to find an optimum condition. Using *I_P_* = 400 µA, a peak *P*_OUT_ of 288 µW and a *V_RECT_* of 2.4 V are extracted with an *R_L_* of 20 kΩ, which corresponds to an efficiency of 78.4%. The *P*_OUT_,_calc_ obtained using Equation (12) is 386 µW, which indicates that the additional loss and control power is 98 µW. Since *P*_OUT_ depends on the PE transducer characteristics and the mechanical vibration [18,19], it is difficult to directly compare *P*_OUT_ performance (See Table 3). However, the conventional FBR can be used as a common reference. Under the same conditions, the maximum power that can be obtained using the FBR is 48.8 µW. The results show that the rectifier using MDDE delivers 5.9 times higher *P*_OUT_ than that of the FBR. Using *I_P_* = 600 µA, a *P*_OUT_ of 441 µW is extracted with a *V_RECT_* of 2.1 V using an *R_L_* of 10 kΩ. The result shows that efficiency is reduced when *I_P_* is increased from 400 to 600 µA. This is related to the narrow working window where *V*_D_ is increased with *I_P_* (See Figure 6).

Table 3 shows a performance comparison with previous works realized using IC technology. The work in [2] presents a resonant rectifier using the bias-flip technique. The bias-flip timing is controlled by a digital inverter delay line that can be programmed externally. Although the adjustable delay control provides flexibility to accommodate various PE transducers, self-startup is not supported. Further, the efficiency of 58% is rather low, which can be attributed to the voltage drop in bias-flip switches and rectifying stages. In [4], a passive differentiator is used to detect the *I_P_* polarity change. In this work, two separate paths are used to reduce the voltage drop for bias-flip. A relatively high *P*_OUT_ of 1230 µW is achieved under externally-powered conditions. The work in [6] proposes a parallel synchronized switch harvesting on inductor (P-SSHI) technique which extracts 48 µW. The result in [20] shows a high efficiency of 91%. Including [20], however, the results in [4,6] are based on discrete realization and not included for comparison.

The work in [21] presents a series synchronized switch harvesting on inductor (S-SSHI) technique, which is applied to the conventional FBR. It shows a good efficiency of 89.5%. However, the results in [3,21] are obtained using an equivalent model of the PE transducer, therefore, its performance in a real environment is unknown. The work in [22] increases power extraction by investing energy from the battery to enhance the electromechanical coupling factor. The work in [23] presents a fully-integrated interface to a PE transducer which does not employ an inductor. By dynamically switching between parallel and series configurations of two PE transducers, this work achieves a peak efficiency of 77%. The work in [24] inserts an active diode in the resonant loop of the SSHI circuit which allows bias-flipping to occur at an optimal time without the need for complicated delay-tuning circuits. In the proposed rectifier using LDDE, we achieve an efficiency of 68.1% under the external-power condition. Under the self-power condition, the rectifier using MDDE delivers a *P*_OUT_ of 288 µW and a *V_RECT_* of 2.4 V. This corresponds to an efficiency of 78.4%. Although this efficiency number is lower than the results of [24], a *P*_OUT_ of 441 µW is extracted using *I_P_* = 600 µA, demonstrating the high power extraction capability of the proposed work. The results indicate the improvement is achieved using efficient bias-flipping with a low *V*_D_ of the MDDE. 

## 4. Conclusions

In this work, we presented an efficient rectifier design technique for PE energy harvesting. To reduce the voltage drop in the rectifier, two diode equivalents are proposed. The diode equivalents are successfully embedded in two resonant rectifiers using the symmetric bias-flip technique. In addition, time synchronization of the bias-flip with the PE transducer is studied. Further, we propose a self-power boosted pulse generator that synchronously detects the zero crossing transition of the PE transducer. The measured results show that the proposed rectifiers significantly increase the extracted power and efficiency. The rectifier using LDDE delivers a *P*_OUT_ of 564 µW with a corresponding efficiency of 68.2%. The rectifier using MDDE delivers a *P*_OUT_ of 288 μW with a peak efficiency of 78.4%. Compared to the conventional FBR, these results show that the rectifier using MDDE and LDDE achieves a power extraction capability enhanced by factors of 5.9 and three times, respectively. The results indicate that an improvement is achieved with the proposed diode equivalents. This result can play a valuable role in various sensing applications that demand energy harvesting to obtain auxiliary power for extended battery lifetime.

## Figures and Tables

**Table 1:** Values of the delay line depending on *R_L_*, *I_P_*, and *f_P_*.

*f_p_* (Hz)		200			300			400		
*R_L_* (KΩ)		100	50	20	100	50	20	100	50	20
*R_DL_* (MΩ)/*C_DL_* (nF)										
*I_P_* (µA)	200	8/1	8/1	10/1	8/1	8/1	8/1	8/1	8/1	8/1
	300	10/1	14/1	12/1	8/1	4/1.5	4/1.5	5/0.5	5/0.5	5/0.5
	400	10/1	14/1	14/1	8/1	8/1	5/1	8/0.5	8/0.5	3/1.2
	600	16/1	16/1	14/1	8/1.5	8/1.5	7/1.5	8/1	8/1	5/1.2

**Table 2:** Parameters for the experiment.

Parameters	Value
*f_p_* *C_p_*	200 Hz 110 nF
*R_p_*	1 MΩ
*L*_*G*1_, *L*_*G*2_, *L*_*G*3_	1000 µH
*C_L_*	1 µF
*R_L_*	10–200 KΩ

**Table 3:** Performance comparison with previous works.

	This Work		[2]	[3]	[21]	[22]	[23]	[24]
	Rectifier (MDDE)	Rectifier (LDDE)						
Tech.	0.18 μm		0.35 μm	0.18 μm	0.18 μm	0.35 μm	0.35 μm	0.25 μm
Type	Bias-flip/diode equivalent		Bias-flip	Active FBR	S-SSHI	Energy Invest	Inductor-less	SSHI
*V_P_* (V)	2.89	4.34	2.8	2.8	2.2	2.6	2.5	4.9
*f*_0_ (Hz)	200		225	200	200	143	82	144
PE transducer	Thrive K7520BP2		V22B Mide	Circuit model	Circuit model	V22B Mide	V22W Mide	V22B Mide
Self-power	Y	N	N	N	Y	N	N	Y
*V_RECT_* (V)	2.4	3.36	3.2	2.78	3.6	3.7	1.0	3.9
*P*_OUT_ (μW)	288	564	53	81	74	52	35	136
Efficiency (%)	78.4	68.1	58	90	89.5	69	77	85
